# Human Hepatocellular Carcinoma (HCC)

**DOI:** 10.3390/cancers12123739

**Published:** 2020-12-12

**Authors:** Luigi Buonaguro

**Affiliations:** Laboratory of Innovative Immunological Models, Istituto Nazionale per lo Studio e la Cura dei Tumori, “Fondazione Pascale”—IRCCS, 80131 Naples, Italy; l.buonaguro@istitutotumori.na.it

Hepatocellular carcinoma (HCC) is the fifth most common cancer and accounts for 8.2% of all cancer-related deaths globally, second only to lung cancer, with more than 800,000 new cases and deaths per year [1].

Surgery (e.g., resection and transplantation) is the most effective treatment for early stage HCC patients on an intention-to-treat perspective, leading to a 60–80% five-year survival. In more advanced disease stages, loco-regional therapies are recommended, with dramatically lower and highly variable survival rates [2]. For many years, the tyrosine-kinase inhibitor Sorafenib has been the only approved first-line systemic therapy for advanced unresectable HCC. Only recently, Lenvatinib, an inhibitor of vascular endothelial growth factor receptors 1–3, has also been approved. Few other drugs have been approved as second-line treatments, but overall, a very limited survival benefit is achieved, unless patients are stratified according to gene expression signature [3,4,5]. Very recently, the IMBRAVE150 trial based on a combination of atezolizumab plus bevacizumab was shown to extend median progression-free survival (PFS) and overall survival at 12 months (OS) compared with Sorafenib [6].

Concerning immunotherapy strategies, namely adoptive T cell therapies (ACTs) and therapeutic cancer vaccines, very few trials have been conducted to date, targeting a limited number of overexpressed target cellular proteins with unsatisfactory results [7]. Among these, only glypican-3 (GPC-3) appears to be highly specific for HCC, with high expression in tumor cells and low or no expression in normal cells [8]. Novel shared HCC-associated antigens have been recently identified within the HEPAVAC project, and a multi-epitope, multi-HLA peptide vaccine is currently being evaluated in a phase I/II clinical trial in an adjuvant setting enrolling early-intermediate stage HCC patients (HepaVac-101-NCT03203005) (www.hepavac.eu) [9,10].

Therefore, HCC remains to date a highly unmet disease, and more insights into different aspects, such as new treatment strategies, the identification of new biomarkers, as well as evaluation of the impact of tumor microenvironment, are needed. The Special Issue on “Human Hepatocellular Carcinoma (HCC)” was launched for providing an update on such different aspects.

An important aspect requiring further development is the reliable early detection of HCC for a timely and optimal treatment. Kim et al. developed a metabolite panel to differentiate early-stage HCC from cirrhosis, identifying five metabolites (methionine, proline, ornithine, pimelylcarnitine, and octanoylcarnitine) that demonstrated a significantly better predictability than Alpha-fetoprotein (AFP) [11].

In the context of HCC treatment, a strategy to improve the efficacy of the anti-angiogenic drug Vandetanib when combined with radiation treatment is proposed by Znati et al. in a preclinical model. Such a combination was assessed in vitro and in a syngeneic mouse model of HCC, showing a significant enhancement of cell killing and inhibition of cell migration and invasion in vitro, as well as reduced cancer growth and improved overall survival in vivo [12]. Xing et al. described an additional innovative therapeutic strategy based on Haprolid, a novel natural component isolated from myxobacteria, which has been shown to be cytotoxic in several tumor cell lines. Haprolid was shown to inhibit cell proliferation, migration, and invasion of several HCC cell lines in vitro through dual inhibition of Rb/E2F and Akt/mTOR pathways, and was also shown to impair the epithelial–mesenchymal transition (EMT). Moreover, in vivo nude mice experiments demonstrated significant inhibition of tumor growth [13]. Both studies provide promising supporting data to be further evaluated in an early phase clinical trial.

Hepatitis C virus (HCV) eradication treatment with the direct antiviral agent (DAA) Sofosbuvir has been associated with HCC lesions’ insurgence, representing a major negative drawback for patients. In order to find a statistical correlation between these two events, Ogawa et al. performed a metanalysis to assess the incidence of HCC after treatments with Sofosbuvir-based or Sofosbuvir-free regimens. Propensity score matched analysis showed that DAA treatment with Sofosbuvir was not associated with the development of de novo HCC within five years. Moreover, the distribution of the early stage of HCC was similar for all treatment regimens, irrespective of the use of Sofosbuvir. Such analysis is extremely relevant in order to remove concerns about the potential dangerous drawbacks of the Sofosbuvir treatment, which has dramatically changed for the better the clinical course of chronic HCV infection [14]. Another important aspect is the identification of members of the HCC patient population that would benefit from treatments. Tampaki et al. showed that the serum levels of T-cell immunoglobulin and mucin domain-3 (TIM-3) is higher in patients with advanced HCC, regardless of the viral etiology. Moreover, higher serum levels were found in patients undergoing transarterial chemoembolization (TACE), and complete responders had higher post-TACE TIM-3 levels than partial responders. These results confirm the importance of patient stratification for improved efficacy of drug treatment. Moreover, they set a basis for combining immunotherapy and chemoembolization [15].

The role of the tumor microenvironment (TME) in the clinical outcome of HCC patients has been addressed by two studies by Takashi et al. and Pascut et al. The first study established a cytolytic activity score (CYT), defined by granzyme A and perforin expression, which was shown to be directly associated with the clinical outcome. CYT-high HCCs were associated with higher expression of gene sets related with enhanced immune activity, supporting a longer disease-specific survival (DSS) and overall survival (OS) [16]. The second study, instead, addressed the cross-talk between tumor cells and those constituting the TME in HCC. In particular, the exosomal microRNAs deliver a whole set of molecules able to regulate cellular pathways in targeted cells related to tumor progression, invasion, and metastasis. Therefore, exosome-derived miRNAs may play a key role in the cross-communication between tumor cells and hepatic resident cells, with clinical implications for immunotherapy strategies and resistance to therapeutic agents [17]. Both studies show the relevance of proper assessment of the TME to stratify HCC patients for appropriate treatments.

Another point of great clinical relevance is the prediction of HCC recurrence after liver transplantation (LT). Chang et al. addressed this aspect by comparing the MoRal score with different criteria currently in place in Eastern and Western countries. The analysis showed that the MoRal score was the best predictor of the risk of tumor recurrence after LT, especially in the sub-cohort of patients beyond the conventional Milan criteria (*p* < 0.001). This represents a valuable information for the follow-up of patients undergoing liver transplantation in order to manage a timely treatment [18].

Overall, the studies published in the Special Issue on “Human Hepatocellular Carcinoma (HCC)” provide results and data of high interest covering different aspects of the management of HCC, representing important reference readings for specialists in the field.
